# Incidence of giant cell arteritis is associated with COVID-19 prevalence: A population-level retrospective study

**DOI:** 10.1016/j.heliyon.2023.e17899

**Published:** 2023-07-03

**Authors:** Ben Mulhearn, Jessica Ellis, Sarah Skeoch, John Pauling, Sarah Tansley

**Affiliations:** a Royal National Hospital for Rheumatic Diseases, Royal United Hospitals Bath NHS Trust, Combe Park, Bath, BA1 3NG, UK; b Department of Life Sciences, The University of Bath, Claverton Down, Bath, BA2 7AY, UK; c North Bristol Hospital NHS Trust, Southmead Hospital, Bristol, BS10 5NB, UK

**Keywords:** Giant cell arteritis, Epidemiology, COVID-19, Vasculitis, Rheumatology, immunology, Pathogenesis

## Abstract

**Background:**

Following the first wave of the COVID-19 pandemic, it was observed that giant cell arteritis (GCA) diagnoses increased at the Royal National Hospital for Rheumatic Diseases (RNHRD) in Bath, UK. This finding may support the viral aetiology hypothesis of GCA. Better understanding of the causes of GCA may help improve diagnostic and treatment strategies leading to better outcomes for patients.

**Objectives:**

The study aims to estimate the local incidence of GCA during the early COVID-19 pandemic (2020–2021) and compare it to pre-pandemic (2015–2019) data. This study will also evaluate the temporal relationship between COVID-19 infections and GCA diagnoses.

**Methods:**

Annual incidence rates of GCA were calculated between 2015 and 2021. Local COVID-19 prevalence was estimated by measuring the number of hospital beds taken up by COVID-19 positive patients. Poisson statistics were used to compare the annual mean incidence of GCA between 2019 and 2020, and Granger causality tested the temporal relationship between COVID-19 prevalence and GCA incidence.

**Results:**

There were 60 (95% confidence interval [CI] 46–77) GCA diagnoses made in 2020 compared to 28 (CI 19–41) in 2019 (*P* = 0.016). Peaks in the number of COVID-19 inpatients correlated with peaks in GCA diagnoses. Granger causality testing found a statistically significant association between these peaks with a lag period of 40–45 days.

**Conclusion:**

The incidence of GCA in Bath was significantly increased in 2020 and 2021 compared to 2015–2019. The lag period between peaks was 40–45 days, suggesting that the COVID-19 virus may be a precipitating factor for GCA. More work is currently underway to interrogate the causal relationship between these two diseases.

## Highlights

• In the first wave of the COVID-19 pandemic there was an increase in GCA cases of 118%.
• The incidence rate of GCA in the pandemic years of 2020 and 2021 was double that of 2015–2019.
• A significant association was seen between COVID-19 and GCA diagnosis with a delay of 40–45 days.
• This study adds further weight to the viral hypothesis of GCA.

## Graphical abstract

**Figure:**
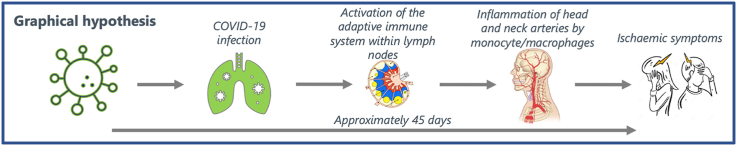

## Abbreviations

CI: Confidence interval
COVID-19: Coronavirus disease 2019
DAMP: Damage-associated molecular pattern
GCA: Giant cell arteritis
GPA: Granulomatosis with polyangiitis
RNHRD: Royal National Hospital for Rheumatic Diseases
RUH: Royal United Hospitals Bath NHS Foundation Trust
TAB: Temporal artery biopsy
TA-USS: Temporal artery ultrasound scan
UK: United Kingdom

## 1 Introduction

Giant cell arteritis (GCA) is a common large vessel vasculitis affecting people over the age of 50. Delays in diagnosis and initiation of treatment can cause serious morbidity including irreversible visual loss [1]. Currently the exact causes of GCA are unknown. It has been postulated that there is an infective aetiology whereby the immune system is sensitised followed by a triggering of the autoimmune response to antigens within large arteries [2]. Previous work has shown that certain infections, particularly viral upper respiratory tract infections, are linked to an increased risk of developing GCA [3]. Better understanding of the relationship between infection and subsequent development of GCA allows the opportunity for enhanced risk stratification, disease recognition and therapeutic options.

Estimates of the incidence of GCA in the UK are between 14.6 and 43.6 per 100,000 annually [4]. During the first wave of the COVID-19 pandemic at the Royal National Hospital for Rheumatic Diseases (RNHRD) in Bath, cases of GCA increased compared to the non-pandemic year of 2019 [5]. Whilst this change was significant, data was only observed over a short period (April–June 2020), therefore warrants further investigation to see if this trend was sustained.

The current study aims to measure changes in GCA incidence rates between the pandemic and pre-pandemic period. We will determine if peaks in COVID-19 prevalence in Bath precede peaks in the incidence of GCA. There have now been at least 2 distinct waves of COVID-19 in the UK allowing statistical analysis to predict GCA cases from COVID-19 prevalence and estimate the delay between COVID-19 infection and diagnosis of GCA.

## 2 Results

### 2.1 Annual incidence of GCA was increased in 2020 and 2021 compared to 2015–2019

We recorded the number of monthly diagnoses of GCA between January 2019 and December 2021, along with the mean bed occupancy of patients at Royal United Hospitals Bath NHS Foundation Trust (RUH) in Bath with confirmed COVID-19 (Table 1).

**Table 1:** Monthly GCA diagnoses between 2015 and 2021 with mean bed occupancy at RUH with COVID-19.

Month	GCA (2015)	GCA (2016)	GCA (2017)	GCA (2018)	GCA (2019)	GCA (2020)	COVID-19 (2020)	GCA (2021)	COVID-19 (2021)
January	4	3	2	2	4	1	0	6	96
February	1	1	4	3	0	2	0	7	55.5
March	3	4	2	4	4	7	20.5	9	12
April	1	4	2	2	2	3	39.5	4	4
May	1	5	1	4	2	12	19	4	1
June	1	0	3	1	1	8	9	7	3.5
July	2	3	3	1	2	2	5	7	12
August	1	4	3	2	5	5	1	9	27
September	1	1	2	3	2	5	1	4	31
October	3	1	2	4	2	6	5	6	41
November	3	1	1	5	4	5	42	3	39
December	2	1	0	1	0	4	60	5	35
Sum	23.0	28.0	23.0	31.0	28.0	60.0	202.0	71.0	357.0
Monthly average	1.9	2.3	1.9	2.6	2.3	5.0	16.8	5.9	29.8

**Fig. 1:**
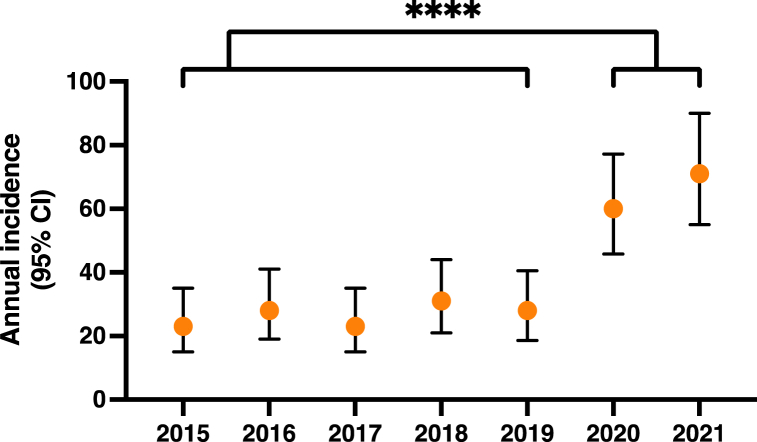
Annual Incidence of GCA at RNHRD between 2015 and 2021. Orange points represent the mean annual incidence of GCA. Lines and error bars represent 95% confidence intervals calculated using the Poisson distribution. *P* value calculated using the unpaired *t*-test with Welch’s correction [****P < 0.0001]. (For interpretation of the references to colour in this figure legend, the reader is referred to the Web version of this article.)

At the RNHRD, the annual incidence rate of GCA between 2015 and 2019 was 26.6 (95% CI 17–39), 60 (95% CI 46–77) in 2020, and 71 (95% CI 55–90) in 2021 (Fig. 1). This represents a significant increase in GCA diagnoses between 2019 and 2020 (P = 0.016), and between 2019 and 2021 (*P* < 0.0001). This is an excess of 33 cases in 2020, or an increase in 118%, alone. Given that 41% of the hospital’s catchment population is over 50, this equates to an annual incidence rate of 13.7 per 100,000 in 2019 (95% CI 7.4–23.1), 29.8 per 100,000 in 2020 (95% CI 20.1–42.6), and 35.0 per 100,000 in 2021 (95% CI 24.4–48.7). This compares to a previously estimated regional incidence rate of 21.6 per 100,000 for the Southwest of the UK [6].

### 2.2 Significant association between peaks in COVID-19 prevalence and GCA incidence with a delay of 40–45 days

**Fig. 2:**
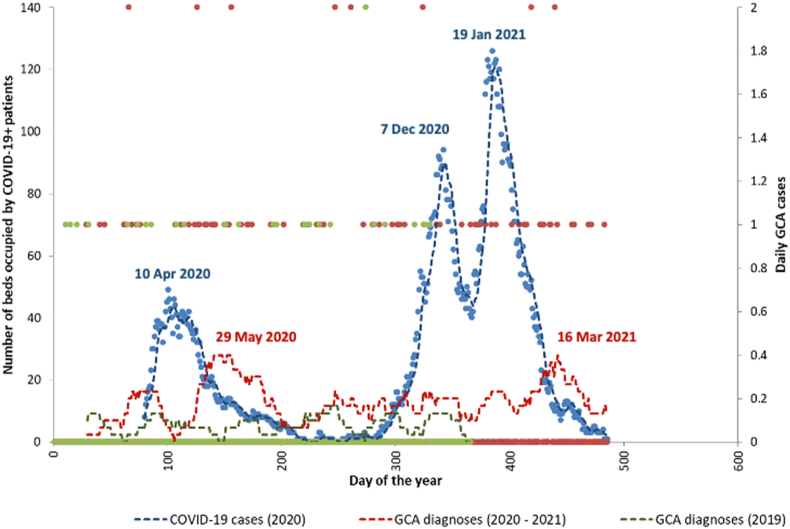
Hospital bed occupancy of COVID-19 and incidence of GCA between 2019 and 2021. Blue dots represent the daily COVID-19 bed occupancy at RUH (left y-axis). Daily GCA incidence from 2020 onwards is shown by red dots (right y-axis) with the 40-day rolling average incidence rate of GCA shown by the red broken line. The green dots and green broken line represent 2019 daily GCA incidence rates (right y-axis). (For interpretation of the references to colour in this figure legend, the reader is referred to the Web version of this article.)

COVID-19 prevalence in the local community was approximated by the number of beds occupied at RUH with COVID-19 positive patients. Distinct peaks in bed occupancy were seen in April 2020, December 2020, and January 2021 (blue dotted line, Fig. 2). Peaks in GCA incidence can be seen following the peaks in COVID-19 in May 2020 and in March 2021.

**Fig. 3:**
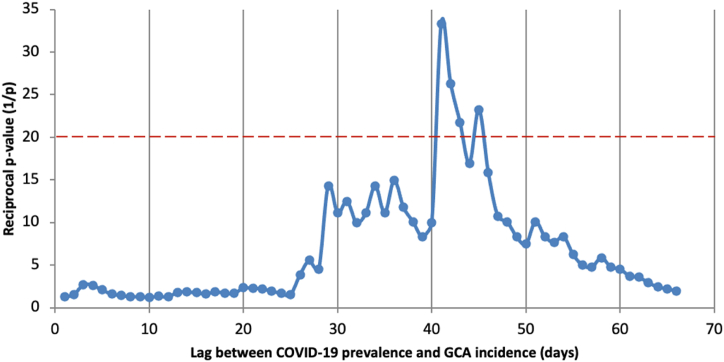
Granger causality testing of incremental lag periods between COVID-19 prevalence and GCA incidence. Blue dots represent each individual reciprocal Granger Causality *P* value (1/*P*). Red dotted line represents a significance level of *P* = 0.05. Any datapoints above the red dotted line represent a significant association between the two time series at those lag points. (For interpretation of the references to colour in this figure legend, the reader is referred to the Web version of this article.)

To test the hypothesis that the COVID-19 prevalence dataset might predict incidence of GCA, the Granger causality test was chosen to test if the COVID-19 time series was able to predict fluctuations in GCA incidence. *P* values above 20 correspond to a *P* value of less than 0.05 (Fig. 3). It was observed that there is a significant association between community COVID-19 prevalence and GCA diagnosis with a lag period of 40–45 days, with the most significant lag period being 41 days (*P* = 0.03).

## 3 Discussion

This study has shown that the incidence of GCA in Bath was significantly higher in the COVID-19 pandemic years of 2020 and 2021 compared to the non-pandemic years between 2015 and 2019. This finding agrees with other groups who found increases in the incidence of GCA, using similar techniques [7,8]. This may be the result of the widespread and uniform infection of a population with the COVID-19 virus as a precipitating factor, as has previously been shown with upper respiratory tract infections [2]. Studies from Scandinavia, where levels of GCA prevalence are amongst the highest in the world, have found that although the prevalence of GCA is decreasing, the incidence of GCA is seasonal with the highest rates in Spring and Summer, which may be suggest a role of prior infection during the Winter months [9]. Although this seasonal effect has been refuted in a large multinational meta-analysis [10], the geographical area studied was Australia and New Zealand, which have different seasonal variations to the Northern Hemisphere and therefore may have different cyclical patterns of infections. To add to the complexity, similarities have been described between the presentation of COVID-19 and GCA which may have artificially increased the diagnosis rate of GCA during the pandemic [11].

By using the Granger causality test [12], this study has established that there is a temporal association between COVID-19 prevalence and GCA diagnosis with a lag period of 40–45 days. Although issues have been raised regarding the validity of this test [13], it nonetheless adds evidence towards an association between the onset of GCA autoimmunity and prior infection. It must be emphasised, however, that causality cannot be inferred from this test alone, despite its name. Interestingly, although not reaching significance, the graph in Fig. 3 shows that there is a weak association between community COVID-19 prevalence and GCA diagnoses with a wider range of 30–50 days. This raises the possibility that, if the hypothesis is valid, there is a degree of inter-individual variability in the latent period between infection and onset of autoimmune disease. This is in line with other groups, who excluded infections within 30 days of GCA diagnosis to minimise classification bias due to similar presenting symptoms [2,3].

One possible mechanism may be the effect of the virus on endothelial dysfunction (as reviewed in Ref. [14]). COVID-19 uses angiotensin-converting enzyme 2. (ACE2) receptor as its co-receptor to enter cells and this receptor is highly expressed on vascular endothelium. Sun exposure has also been proposed as a cause of endothelial dysfunction, with disruption of the internal elastic lamina [15]. Disruption of the internal elastic lamina, which is adjacent to the adventitia within the arterial wall, may release damage associated molecular patterns (DAMPs) to activate adventitial dendritic cells [16], thereby priming naïve T cells towards both T helper cell type 1 (Th1) and Th17 immunity [17]. Both Th1 and Th17 responses are recognised as important in the initiation of autoimmunity [18,19]. Chemokine release from Th1 and Th17 cells within arterial walls then recruits monocytes which differentiate into macrophages, leading to the typical granulomatous lesions seen in GCA [20].

This study has several limitations. Firstly, this is a single-centre study and has not validated using an independent dataset. Any findings may therefore be centre-specific and it would be difficult to generalise the conclusions. To address this, research is currently underway on a large Primary Care database to attempt to validate and build on these findings. Secondly, this is an observational study, and it is therefore possible that unmeasured confounders may have led to the results reported in this study. For example, there may be a supra-annual periodicity to GCA incidence due to an unknown factor which was not appreciated by the short follow time in this study. Granulomatosis with polyangiitis (GPA), another form of vasculitis, is known to have a variable incidence rate with a cycle every 7.6 years [21], which may also be true of GCA. Furthermore, it could be possible that peaks in the incidence of GCA were higher after peaks in COVID-19 prevalence due to delays in health-seeking during heights of the pandemic. For instance, patients may have delayed seeking medical attention during lockdown periods, when governmental advice was to isolate and to only seek medical attention for emergencies. This behaviour change may have artefactually increased the incidence of GCA as COVID-19 lockdown advice eased and patients began to seek medical attention for their symptoms. However, the increase in the annual incidence rate of GCA in 2020 and 2021 compared to the prior years does not agree with this hypothesis.

Access to healthcare providers was more challenging during the pandemic due to staff and resource re-deployment. However, our GCA referral service did not change during the pandemic, with patients seen by specialists within our service as before. Additionally, reduced access to services would make it more likely that GCA cases were under-diagnosed rather than over-diagnosed. Therefore, differential access to services during the pandemic is unlikely to have caused an overestimate of incidence rates.

GCA is a clinical diagnosis with no single test leading to diagnosis. Temporal artery biopsy (TAB) is still perceived as the gold standard technique for diagnosing GCA. During the pandemic, our centre had significantly limited access to TAB, and therefore it is possible that cases were over-diagnosed leading to inflated incidence rates. However, pre-pandemic, our centre already had limited access to TAB relying heavily on temporal artery ultrasound (TA-USS) scanning. Access to TA-USS did not change significantly during the pandemic therefore effect this did not represent a large deviation in practice for our centre. Finally, the symptoms of long-COVID may mimic many of the symptoms of GCA which could potentially lead to overdiagnosis of GCA. However, there are clear clinical differences between these two conditions, e.g., presence and absence of raised inflammatory markers on blood tests in GCA and long COVID respectively, that allow these conditions to be distinguished in practice.

## 4 Conclusions

Giant cell arteritis is an autoimmune disease of unknown aetiology. The COVID-19 pandemic represented a unique opportunity to study the temporal relationship between infection and autoimmunity. This study has found an association between the two conditions on a population level with a lag period of 40–45 days, which may be evidence supporting the viral aetiopathogenesis of GCA. We next aim to investigate this relationship in a national database on an individual patient level. Understanding risk factors for disease will facilitate earlier diagnosis and reduce the serious complications of GCA.

## 5 Materials and methods

### 5.1 Data collection

Routinely collected clinical data was collated from the RNHRD between January 2019 and May 2021 using the hospital’s electronic patient record and through the unified email system where all GCA referrals are received. Patients referred to the fast track GCA service are assessed within 3 days of primary care referral and have baseline inflammatory markers measured (C reactive protein, plasma viscosity, and full blood count to measure platelet count). All patients have TA-USS before assessment. Diagnosis is based on the clinical features of GCA with supporting raised inflammatory markers and a positive TA-USS. If TA-USS did not corroborate the diagnosis of GCA then a temporal artery biopsy was requested. Ultimately, the diagnosis of GCA was confirmed by a Consultant Rheumatologist at 6 months with the benefit of blood testing, imaging, biopsy results, and disease course, to ensure a high true positive rate.

Prevalence of community COVID-19 was estimated using the daily number of beds occupied by COVID-19 patients at RUH, publicly available at [22].

All data was fully anonymised, non-identifiable, and collected as part of routine practice at the RNHRD. Furthermore, no data was made available outside the team providing direct clinical care. After consulting the Health Research Authority and the local Research & Development Team, it was felt that ethical approval was not necessary via an NHS Research and Ethics Committee.

### 5.2 Statistical analysis

Annual incidence of GCA was assumed to follow the Poisson distribution and 95% confidence intervals were calculated using this distribution. Unpaired parametric t-testing with Welch’s correction for non-uniform variance was used to compare the monthly incidence rates of GCA between 2019 and 2020 using the software GraphPad Prism version 9.4.1 for macOS.

Analyses were performed using RStudio (2022.07.2 Build 576) [23] with R Statistical Software (v4.2.2) [24]. Granger causality testing assessed the relationship between daily COVID-19 bed occupancy and daily GCA incidence. Briefly, Granger testing generated an array of *P* values using lag periods from 1 to 65 days. The inverse of the *P* value array was then plotted to observe any significant association according to lag. The grangertest() function of the R package lmtest [25] generated a table of *P* values using lag periods from 1 to 65 days. Reciprocal *P* values were plotted using Microsoft Excel version 16.67 for Mac.

## Data statement

All anonymised raw data collected has been deposited in the Mendeley Data Repository [26].

## Author contribution statement

All authors conceived and designed the study, and contributed to analysing and interpreting the data. BM wrote the first draft of the paper and all authors contributed to the significantly to the final drafts of the paper.

## Funding statement

BM is an NIHR-funded Academic Clinical Fellow (ID SEV/008-001/7018282/C).

## Declaration of competing interest

The authors declare that they have no known competing financial interests or personal relationships that could have appeared to influence the work reported in this paper
